# An Exploratory Study of the Impact of a CCL21‐Derived C‐Terminal Peptide on Dendritic Cell Lymph Node Homing

**DOI:** 10.1155/jimr/8833079

**Published:** 2026-01-05

**Authors:** Marina Barrio-Calvo, Astrid Sissel Muldorff Frellsen, Emma Probst Brandum, Mette Marie Rosenkilde, Eoghan Connors, Per Basse, Pawel Kalinski, Gertrud Malene Hjortø

**Affiliations:** ^1^ Department of Biomedical Sciences, Faculty of Health and Medical Sciences, University of Copenhagen, Copenhagen, Denmark, ku.dk; ^2^ Evaxion A/S, Hørsholm, Denmark; ^3^ Department of Pharmacology and Therapeutics, Roswell Park Comprehensive Cancer Center, Buffalo, New York, USA, roswellpark.org

**Keywords:** CCR7, dendritic cells, dendritic-cell migration, lymph node homing

## Abstract

The effective trafficking of dendritic cells (DCs) to the lymph nodes (LNs), orchestrated by CC‐chemokine receptor 7 (CCR7) and its ligand CCL21, is essential for the success of DC–based immunotherapies. This study explores the potential of C21TP, a naturally occurring basic peptide derived from the C‐terminal of CCL21, to enhance DC homing to the draining LNs in a murine model of DC migration. C21TP, containing three clusters of basic residues, significantly boosts CCL21‐mediated signaling and chemotaxis of DCs in vitro. In vivo, DCs formulated with C21TP prior to injection migrated more efficiently to the draining LNs than DCs alone or DCs formulated with a mutated version of C21TP, harboring substitutions in key basic residues. Further studies are needed to evaluate the impact of C21TP on T‐cell priming efficacy in the context of DC–based immunotherapies. Nonetheless, C21TP’s ability to enhance lymph node homing of adoptively transferred cells without additional cellular modifications could offer a practical and scalable approach for advancing future DC–based vaccines.

## 1. Introduction

The efficient trafficking of immune cells to lymph nodes (LNs), orchestrated by the chemokine system, is a critical determinant of the success of adoptive cell transfer immunotherapies, which aim to reinvigorate the patient’s immune system by reinfusing immune cells, including dendritic cells (DCs) and T‐cells [1–3]. Strategies to improve migration often involve genetically modifying the infused cells to express chemokines and their receptors, or preconditioning the injection site with inflammatory cytokines [4–6]. The CC‐chemokine receptor 7 (CCR7) and its two ligands, CCL19 and CCL21, are critical for the migration of antigen‐loaded DCs to the LNs, where adaptive immune responses are initiated [7]. Therefore, engineering DCs to express CCR7 or CCL21 has been explored to improve the efficacy of DC–based therapies in preclinical and clinical settings [5, 8, 9].

Naturally occurring positively charged peptides have been recently identified as modulators of chemokine–receptor interactions, expanding an already complex regulatory landscape [10, 11]. Signaling through CCR7 is regulated by the naturally occurring peptide C21TP, derived from the proteolytic cleavage of the C‐terminal of CCL21 [12, 13]. DC‐released proteases and plasmin cleave CCL21 to generate a shorter chemokine (tailless or soluble CCL21) lacking the basic C‐terminus and the basic C‐terminal‐derived peptides [14, 15]. The peptide under investigation in the current study, CCL21^81–111^, hereafter referred to as C21TP, spans amino acids 81–111 of CCL21 (Figure 1A). C21TP contains three clusters of basic residues (two BBxB and one BBxxB motifs) that bind to the negatively charged N‐terminal region of CCR7 [12, 13]. In vitro, the electrostatic interactions between C21TP and CCR7 enhance the signaling efficiency of the full‐length CCL21, resulting in improved DC chemotaxis towards CCL21 gradients [12, 13].

Figure 1The Basic domains in C21TP are essential for enhancing CCL21–CCR7 signaling and chemotactic responses of murine DCs. (A) Peptide sequences of C21TP and its mutated derivative mut‐C21TP. Basic residues are highlighted in red, BBxB and BBxxB motifs are annotated, and glutamine substitutions are highlighted in bold. (B) Effect of 10 *μ*M of C21TP and mut‐C21TP in combination with different CCL21 concentrations ranging from 0 to 100 nM on the G*α*i signaling of CHO‐K1 cells transfected with CCR7. Signaling is evaluated with a BRET‐based assay. Data are presented as mean ± SEM (*n* = 3–4 independent experiments). (C) Schematic representation of the experimental workflow used to generate exogenous DCs and flow cytometry analysis of their phenotype. CD45.1 donor mice were injected subcutaneously with B16‐FLT3L tumor cells to promote the expansion of DC progenitors. Fourteen days later, CD11c^+^ progenitor DCs were isolated from the spleens using magnetic beads. DCs were differentiated overnight using GM‐CSF, and further activated for 4 h using a cytokine cocktail containing GM‐CSF, TNF‐*α*, IFN‐*γ*, IL‐1*β*, IFN‐*α*, and Poly I:C. Flow cytometry analysis of naïve splenocytes and the isolated DCs at the different stages of the maturation process showed surface expression of lineage and co‐stimulation markers (CD11c, CD11b, MHC‐II, XCR1, CCR7, and CD86). (D) The migration ability of murine DCs cells in response to 10 nM CCL21 alone or in combination with 10 *μ*M C21TP and mut‐C21TP was assessed using a 3D chemotaxis assay. (D1) DC migration quantification represented as chemotactic index (mean ± SEM; *n* = 3–5 independent experiments). (D2) Spider diagrams represent migration tracks of individual cells towards the chemoattractant source, which is placed on the left. Distances are depicted in *μ*m. Statistics: (B) Ordinary two‐way ANOVA and Tukey’s multiple comparison tests (simple effects within ligand concentration). Comparisons of statistical significance against the CCL21 alone group are displayed. (D1) Ordinary one‐way ANOVA and Tukey’s multiple comparison test.  ^∗∗^ *p* < 0.01,  ^∗∗∗^ *p* < 0.001,  ^∗∗∗∗^ *p* < 0.0001.

**Figure:**
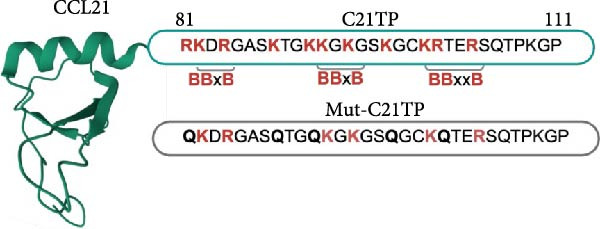
(A)

**Figure:**
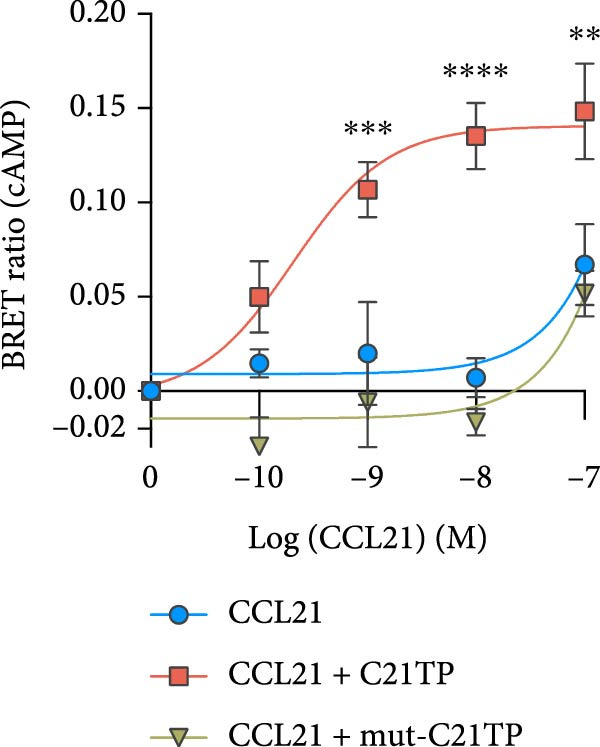
(B)

**Figure:**
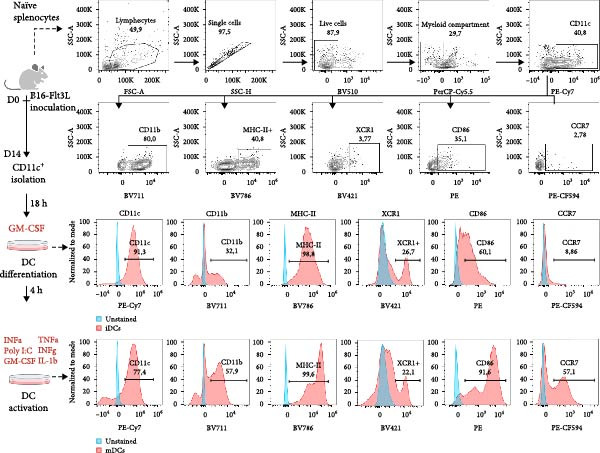
(C)

**Figure:**
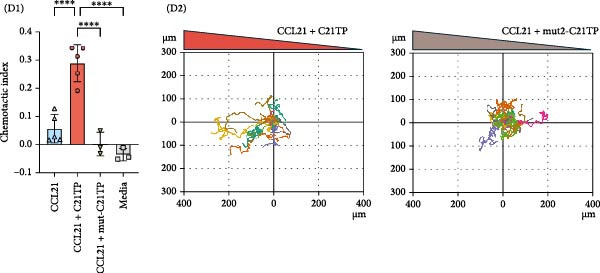
(D)

This study provides the first in vivo evidence indicating that formulating ex vivo–activated DC with C21TP improves their homing to draining LN in an animal model, supporting its applicability as an immune cell migration modulator. The potential use of C21TP as an exogenous bioactive enhancer of CCR7–mediated migration represents a practical and scalable approach for improving immune cell trafficking and advancing cell‐based immunotherapies.

## 2. Methods

### 2.1. Cell Lines

The B16‐FLT3L melanoma cell line originating from a male C57BL/6 mouse (RRID: CVCL_IJ12) was kindly provided by Pawel Kalinski, Roswell Park Comprehensive Cancer Center, USA. At the time of acquisition, the B16‐FLT3L cell line was subjected to IDEXX BioAnalytics IMPACT profile testing to confirm the absence of rodent pathogens. The CHO‐K1 cell line (RRID: CVCL_0214) originates from the ovary of an adult female Chinese hamster and was originally obtained from ATCC, USA (#CCL‐61). Both cell lines are regularly tested for mycoplasma to confirm the reliability of the experiments and are not listed in the ICLAC register of misidentified or contaminated cell lines. Both cell lines were kept at 5% CO_2_, 37°C in RPMI supplemented with 10% fetal bovine serum (FBS), 2% penicillin/streptomycin (P/S), and 2% L‐glutamine.

### 2.2. Basic Peptides

Basic peptides were produced by Caslo (Denmark), dissolved in PBS, and stored at −80°C. C21TP (RKDRGASKTGKKGKGSKGCKRTERSQTPKGP) corresponds to the last 32 amino acids of human CCL21. Mut‐C21TP (QKDRGASQTGQKGKGSQGCKQTERSQTPKGP) is generated by substituting arginine (R) and lysine (K) residues in the C21TP sequence with glutamine (Q). Substitutions on the sequences are marked with bold letters.

### 2.3. CCR7 G*α*i Signaling

CCR7 signaling was evaluated in adherent CHO‐K1 cells using a bioluminescence resonance energy transfer (BRET) assay to quantify changes in intracellular cAMP downstream ligand‐induced signaling [16]. CCR7 signals via G*α*i, which inhibits adenylate cyclase, thereby lowering intracellular cAMP levels upon ligand interaction. In brief, 500,000 cells/well were seeded in six‐well plates and transiently co‐transfected with the human CCR7 vector and the CAMYEL sensor (a cAMP sensor using YFP‐Epac‐RLuc) using Lipofectamine 2000 (11668027, ThermoFisher) according to the manufacturer’s instructions. The following day, the cells were resuspended in PBS with glucose and formulated with C21TP peptides (10 µM), seeded in 96‐well white iso plates (~25,000 cells/well) and treated with coelenterazine (3551, Nanolight) to a final concentration of 5 µM. After 10 min, cells were stimulated with varying concentrations of CCL21. Forskolin (F6886, Sigma) was added to each well 5 min after CCL21 to a final concentration of 5 µM. Forskolin stimulates adenylate cyclase, leading to high intracellular levels of cAMP, which allows for the quantification of CCR7 activation as a ligand‐induced decrease in cAMP. The emission signals from Rluc and eYFP were measured using the Envision plate reader at 530 and 480 nm 35 min after CCL21 addition. The BRET signal was determined as the ratio between eYFP and Rluc [12].

### 2.4. Animals

Six to 12‐week‐old C57BL/6J and B6.SJL‐*Ptprca Pepcb*/BoyCrl female mice were acquired from Charles River. Animal experiments were performed under the licenses 2020‐15‐0201‐00618 and 2023‐15‐0201‐01590 from the Danish Animal Experiments Inspectorate and the local ethical committee in accordance with the Danish Animal Experimentation Act BEK nr. 12 of 7/01/2016), which is compliant with the European directive (2010/63/EU).

### 2.5. Murine Dendritic Cell Expansion, Harvest, and Maturation

#### 2.5.1. Tumor Cell Inoculation

CD45.1 expressing B6.SJL‐*Ptprca Pepcb*/BoyCrl mice were inoculated subcutaneously (s.c.) in the neck with 1 × 10^6^ B16‐FLT3L tumor cells in 50 μL. Tumor volume was monitored twice per week. The experiments were terminated 14 days after tumor inoculation or upon humane endpoints (12 mm tumor diameter in any direction or tumor ulcerations).

#### 2.5.2. Spleen Harvest

Mice were euthanized by cervical dislocation, and spleens were harvested in RPMI 1640 media supplemented with 2% FBS and 1% P/S and processed into single‐cell suspensions by grinding through a 70 and a 40 µm filter, followed by incubation with RBC lysis (00‐4333‐57, Invitrogen) for 15 min at room temperature.

#### 2.5.3. Dendritic Cell Isolation and Differentiation

CD11c+ positive DCs were isolated from splenocytes using magnetic beads (18780, STEMCELL) according to the manufacturer’s instructions and cultured overnight at 5% CO_2_, 37°C in DC culture media supplemented with murine GM‐CSF (1 × 10^3^ U/mL, 315‐03, Peprotech) as previously described [17]. DC culture media contains 50% RPMI 1640 with L‐Glutamine medium, 50% AIMV medium (12055083, ThermoFisher), 10% FBS, 1% sodium pyruvate (11360‐070, Gibco), 1% MEM nonessential amino acids, 1% HEPES buffer, 1% P/S, and 0.0001% 2‐Mercaptoethanol (M3178, Sigma).

#### 2.5.4. Dendritic Cell Maturation

After overnight culture with GM‐CSF, DCs were activated as previously described [17]. I brief, DCs were cultured for 4 h at 5% CO_2_ 37°C in DC culture media supplemented with murine GM‐CSF (1 × 10^3^ U/mL, 315‐03, Peprotech), murine TNF‐*α* (5 ng/mL, 315‐01A, Peprotech), murine interferon *γ* (IFN‐*γ*; 5 × 10^2^ U/mL, 315‐05, Peprotech), murine IL‐1*β* (25 ng/mL, 211‐11B, Peprotech), murine IFN‐*α* (1 × 10^3^ U/mL, 12100‐1, R&D systems), and Poly I:C (100 µg/mL, P9582, Merck). After maturation, the cells were frozen in FBS supplemented with 10% DMSO.

### 2.6. Phenotypic Characterization of Ex Vivo Activated DCs by Flow Cytometer

To confirm the correct expression of DC activation markers after DC isolation and maturation, naïve splenocytes, and CD11c^+^ DCs at different stages of the activation process were stained with fluorochrome‐labeled antibodies, including viability dye (GloCell Fixable Viability Dye, 75010, Stem Cell Tech), PerCP‐Cy5.5 anti‐CD3 (551163, BD), PerCP‐Cy5.5 anti‐CD19 (152405, Biolegend), BV786 anti‐MHC‐II (743875, BD), PE‐Cy7 anti‐CD11c (561022, BD), BV711 anti‐CD11b (563168, BD), BV421 anti‐XCR1 (148216, Biolegend), PE anti‐CD86 (105007, Biolegend), and PE‐CF594 (563596, BD). Stained cells were acquired in a CELESTA flow cytometer (BD) and analyzed using the commercial software FlowJo. Gates are established based on fluorescence minus one (FMO) controls.

### 2.7. Three‐Dimensional (3D) Chemotaxis Assay

The effect of C21TP on the migration of murine DCs towards CCL21 was assessed in a 3D chemotaxis assay as previously described [13]. In brief, upon thawing, DCs were embedded in a collagen‐I matrix and loaded into µ‐Slide Chemotaxis chambers (80326, Ibidi) where they were allowed to migrate towards a chemotactic gradient of 10 nM CCL21 alone or in the presence of 10 μM of C21TP or mut‐C21TP. Cell migration was monitored using video microscopy over 16 h at 2‐min intervals in a time‐lapse microscope equipped with a humidified and temperature‐controlled stage incubator. Cell migration was tracked using the commercial tracking program Autozell and subsequently analyzed to get a population‐based chemotactic index (CI) value using the MatLab software. CI is calculated as the ratio of the distance traveled in the direction of the gradient over the total distance traveled. Cell movement and migration distance (μm) were visualized in spider diagrams created using the Python software.

### 2.8. Injection of Ex Vivo Activated DCs and Analysis of In Vivo Migration

Frozen CD45.1 CDs were thawed in RPMI + 10% FBS, washed, and acclimatized for 20 min at 5% CO_2_, 37°C. DCs were then counted, spun, resuspended in PBS at 21.1 × 10^6^ cells/mL and aliquoted to be formulated with C21TP, mut‐C21TP or PBS. The peptides C21TP or mut‐C21TP (200 μM stock), were added to the DCs at a final concentration of 10 μM resulting in a final cell concentration of 20 × 10^6^ cells/mL. The DCs were incubated at room temperature for 30–60 min. The peptides are not washed prior to injection. A total of 0.3 × 10^6^ CD45.1 expressing DCs were injected s.c. into each of the hocks or footpads of CD45.2 C57BL/6J mice. When C21TP is injected alone in the absence of DCs, it is diluted in PBS to 10 μM and administered s.c. (15 µL) in the hock.

24 h after injection, the popliteal LNs (pLNs) were harvested in RPMI 1640 media supplemented with 2% FBS and 1% P/S and processed into a single‐cell suspension by grinding through a 40 µm filter. Unpublished data revealed that injected cells do not reach the inguinal LN within 24 h. Therefore, only the popliteal LN is presented in this report. The isolated cells were blocked with FC blocker (14016182, Biolegend) for 5 min before being stained at 4°C for 30 min with PE anti‐CD45.1 (110707, Biolegend), BV510 anti‐MHC‐II (107636, Biolegend), and FITC anti‐CD45.2 (561087, BD). After staining, the cells were fixed in 1% paraformaldehyde overnight and washed in PBS + 2% FBS prior to acquisition in a CELESTA flow cytometer (BD) and analyzed using FlowJo. Each pLN (two per mouse) is treated as an experimental unit.

### 2.9. Statistics

GraphPad Prism 9 for Mac OS X was used for graphical representations and statistical analyses. Due to the limited sample sizes, a robust assessment of normality was not possible. Consequently, parametric tests were employed to evaluate statistical differences among the experimental groups. Dose‐response curves were analyzed using ordinary two‐way ANOVA, with simple effects within ligand concentration chosen for multiple comparisons. Tukey’s test was applied to correct *p*‐values for multiple comparisons. Mean comparisons across multiple groups were analyzed using one‐way ANOVA, followed by Tukey’s test for multiple comparisons. While all possible comparisons were conducted and the *p*‐values appropriately corrected, only those relevant to the study’s objectives are displayed in the figures. Statistical significance was defined as follows: ns *p* ≥ 0.05,  ^∗^ *p* < 0.05,  ^∗∗^ *p* < 0.01,  ^∗∗∗^ *p* < 0.001,  ^∗∗∗∗^ *p* < 0.0001.

## 3. Results

### 3.1. The Basic Domains in C21TP Are Important to Boost CCL21–CCR7 Signaling and Migration In Vitro

DC chemotaxis towards CCL21 gradients is regulated by the interaction of the naturally occurring peptide C21TP with the negatively charged N‐terminal region of the CCR7 receptor. This electrostatic interaction is mediated by three BBxB/BBxxB domains in C21TP.

To confirm that the basic motifs of C21TP are critical to its function, we designed the control mutated peptide mut‐C21TP in which basic residues in the BBxB and BBxxB motifs are substituted with neutral amino acids (Figure 1A). In a BRET‐based assay using CCR7‐transfected CHO‐K1 cells, C21TP significantly boosted CCR7‐G*α*i signaling measured as changes in intracellular cAMP in response to different concentrations of full‐length CCL21 (0–100 nM) while mut‐C21TP failed to do so (estimated half‐maximal effective concentration eECM50 = 3.57 × 10^−5^ ± 3.2 × 10^−5^ M vs 2.42 × 10^−10^ ± 8.99 × 10^−11^ M). On their own, none of the peptides triggered CCR7 signaling (Figure 1B). These findings underscore the critical role of C21TP’s basic domains in boosting CCL21‐induced CCR7 signaling.

To assess the potential of C21TP to boost migration of adoptively transferred DCs in a murine model relevant to DC–based immunotherapy, we isolated murine DC progenitors from the spleens of CD45.1^+^ donor mice based on CD11c expression. These cells were then differentiated and matured using a cytokine cocktail previously described to license the cells to initiate a type 1‐polarized immune T cell response (Figure 1C). To obtain a high yield of DC progenitors, CD45.1^+^ donor mice were pretreated with FLT3L‐secreting B16 syngeneic tumor cells, as FLT3L abundance significantly expands DC populations in vivo [18].

Following DC maturation, we confirmed their phenotypic integrity by flow cytometry, showing the presence of both conventional DC1s (MHC‐II^+^ XCR1^+^ CD11b^−^) and conventional DC2s (MHC‐II^+^ XCR1^−^ CD11b^+^). The expression of CD86 and CCR7 markers confirmed DC activation (Figure 1C). Time‐lapse microscopy confirmed DC chemotaxis toward CCL21 when co‐incubated with C21TP but not mut‐C21TP or CCL21 alone, confirming that C21TP enhances CCL21‐induced chemotaxis via its basic residue motifs (Figure 1D). These findings confirm the efficient maturation of DCs into a cell phenotype relevant for studying the effect of C21TP on their migration capabilities in vivo.

### 3.2. The C21TP Peptide Modulates the Homing of Ex Vivo‐Activated DCs to Draining LNs In Vivo

**Figure 2:**
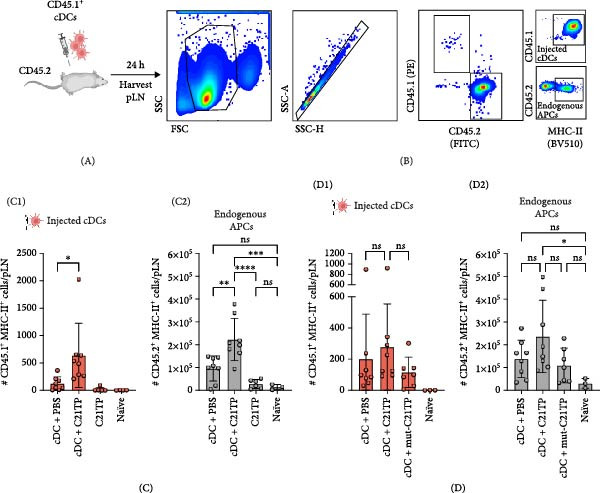
C21TP enhances the homing of dendritic cells to draining lymph nodes. (A) Schematic representation of the in vivo experimental setup used in two independent experiments (C and D). CD45.1^+^ conventional DCs formulated with C21TP, mut‐C21TP, or PBS were injected into the hock of CD45.2^+^ recipient mice. After 24 h, the pLNs were harvested and analyzed by flow cytometry. (B) Flow cytometry gating strategy used to distinguish injected CD45.1^+^ DCs from endogenous CD45.2^+^ immune cells. Cells were gated on live lymphocytes, followed by discrimination of CD45 isoforms, and further validated by MHC‐II expression. (C) Quantification of migrated CD45.1^+^ DCs (C1) injected in combination with C21TP or PBS and endogenous professional APC population, characterized by the expression of CD45.2^+^ and MHC‐II+ (C2). The same gating strategy was used in the pLN of mice injected with C21TP alone. (D) Quantification of migrated CD45.1^+^ DCs (D1) injected in the hock in combination with C21TP, mut‐C21TP, or PBS and endogenous CD45.2^+^ MHC‐II + immune population (D2). Statistics: Data are presented as mean ± SD (*n* = 2–8 individual pLN per treatment group). Subparts (C, D) represent two independent experiments. Ordinary one‐way ANOVA and Tukey’s multiple comparison test. *p*: *p*‐values  ^∗^ *p* < 0.05,  ^∗∗^ *p* > 0.01,  ^∗∗∗^ *p* < 0.001,  ^∗∗∗∗^ *p* < 0.0001.

To assess the effect of C21TP on LN homing, ex vivo‐generated CD45.1^+^ DCs formulated with C21TP, mut‐C21TP, or PBS were injected subcutaneously into the hock of CD45.2^+^ C57BL/6 mice (Figure 2A,B). The presence of migrated cells in the popliteal draining LNs was analyzed by flow cytometry 24 h after injection using fluorescent antibodies for CD45.1, CD45.2, and MHC‐II. The combination of CD45.1 and MHC‐II identifies the injected DCs, while CD45.2 and MHC‐II mark the endogenous antigen‐presenting cells (APCs).

Flow cytometry analysis revealed improved homing of the DCs formulated with C21TP, with a mean of 670 CD45.1^+^ cells/pLN, compared to 124 cells/pLN for DCs formulated with PBS (Figure 2C, C1). Corroborating these results, more DCs migrated to the pLN in the presence of C21TP when injected into the footpads, as both the footpad and the hook share similar lymphatic drainage pathways (Supporting Information: Figure S1).

Interestingly, the lymphatic endogenous APCs population, defined as CD45.2^+^ MHC‐II^+^ cells, increased in mice receiving DCs + C21TP (2.2 × 10^5^ cells/pLN) compared to mice receiving DC1s + PBS (0.96 × 10^5^ cells/pLN) (Figure 2C, C2). The increase correlates with a higher number of translocated, injected DCs, a phenomenon previously described as promoting local LN congestion [19]. Notably, C21TP alone did not alter the numbers of endogenous APCs reaching the pLN. Thus, it is likely that the injection of exogenous DCs acts as an adjuvant, causing activation of endogenous immune cells whose chemotaxis towards the pLN may be susceptible to modulation by small peptides such as C21TP.

Finally, formulating DCs with mut‐C21TP prior to adoptive transfer showed reduced migration efficacy compared to DCs + C21TP and was no different from the DCs + PBS control (Figure 2D, D1). As observed previously, injection of DCs + C21TP demonstrated a trend of increased migration compared to the PBS group, although high data variability prevented statistical significance. Nonetheless, these results confirm that the basic residues in C21TP are essential for enhancing CCR7‐mediated migration. The endogenous APCs reaching the LN did not increase in mice treated with mut‐C21TP, which could indicate that C21TP acts on this population in combination with the adjuvanted effect of injecting exogenous cells (Figure 2D, D2).

## 4. Discussion

This study provides the first evidence of the potential of C21TP, a naturally occurring basic peptide derived from CCL21, as a novel compound for enhancing CCR7‐mediated DC trafficking in vivo. These results align with the emerging role of basic peptides as modulators of chemokine–receptor interactions, expanding the tools for manipulating immune cell migration and potentially improving the efficacy of DC–based immunotherapies.

The enhanced CCR7 G*α*i signaling obtained with C21TP confirms the peptide’s capacity to boost cellular responses to low concentrations of CCL21. Notably, the lack of boosting with the mutated version of C21TP, featuring alterations in key basic residues, confirms the proposed mechanism by which electrostatic interactions between C21TP and the negatively charged N‐terminal domain of CCR7 facilitate the transition of CCL21 from a low to a high affinity electrostatic binding mode, engaging the receptor binding pocket [12]. These findings contribute to expanding the mechanistic framework of CCR7–CCL21 interactions.

The results obtained in the murine DC migration model show that C21TP improves the homing of the injected cells to the draining LNs, highlighting C21TP’s capacity to modulate CCR7‐mediated trafficking also in vivo.

The presented data constitute a first step towards understanding the potential role of C21TP in the migration of injected DC. Nevertheless, several important questions remain, including the extent to which C21TP contributes to the immunogenicity and functionality of DC vaccines, as well as mechanistic aspects such as its influence on endogenous CCL21 displacement from heparan sulfate proteoglycans and its subsequent effect on immune cell migration [12]. Future investigations should assess the impact of co‐administering C21TP with established adjuvants or antigens on the breath and type of the elicited immune responses in order to determine whether this bioactive peptide could represent a feasible approach to optimizing the therapeutic efficacy of cell‐based immunotherapies without the need for genetic alteration of the injected cellular products or preconditioning of the immunization site. Moreover, it will be important to determine whether other basic peptides containing BBXB motifs and known to modulate CCL21–CCR7 interactions in vitro similarly affect the migratory behavior of injected DCs.

We observe higher LN cellularity upon injection of DCs formulated with C21TP, evidenced by the accumulation of CD45.2^+^ MHC‐II^+^ cells in the draining LN. These markers encompass multiple immune populations, including DCs, B cells, and macrophages; however, precise identification was not possible in this dataset due to the absence of lineage‐specific markers such as CD19 for B cells or CD11b for macrophages. These findings align with previous reports linking LN congestion to the number of injected DCs reaching LN [19], which is higher when the injected DCs are formulated with C21TP. In accordance with this observation, the injection of C21TP alone did not exert an increase in cellularity in the LNs. However, given the differences in formulation and the likely short half‐life of free C21TP in vivo, our data do not rule out potential adjuvant properties of C21TP if it were to be included in other therapeutics. While we only interrogated CD45.2^+^ MHC‐II^+^ cells, it is important to note that CCR7 is expressed in other immune cell types that may respond to the enhanced chemotactic signaling induced by C21TP. The potential co‐recruitment of these cells to the LN could enhance interactions necessary for T‐cell priming. However, it also highlights the need to assess how C21TP impacts antigen‐specific immune responses.

In conclusion, the presented data demonstrate that C21TP promotes the homing of adoptively transferred DCs to draining LNs through CCR7. This effect is mediated by clustered basic residues within C21TP, consistent with the in vitro observations. These findings establish C21TP as a practical, nongenetic strategy for optimizing cell‐based immunotherapies by improving immune cell trafficking. C21TP may also modulate chemokine–receptor interactions in other therapeutic contexts where it could act as an adjuvant. Furthermore, the translational confirmation provided here could lead to the assessment of the therapeutic potential of alternative naturally occurring basic peptides, whose ability to modulate cytokine–receptor pathways is already under investigation.

## Ethics Statement

The study was conducted according to the guidelines of the Declaration of Helsinki and approved by the local ethics committee at the Faculty of Health and Medical Sciences at the University of Copenhagen (Research Ethics Committee for Sund and Science, University of Copenhagen) (Protocol Code 504‐0270/21‐5000, approved October 2021).

## Conflicts of Interest

Marina Barrio‐Calvo is employed at Evaxion A/S. Pawel Kalinski has been identified as the inventor of *α*DC1s, one of the models used in the paper. All other authors declare no conflicts of interest.

## Funding

This project has been partially funded by Innovation Fund Denmark via an Industrial PhD grant (Grant 1044‐00028B to Birgite Rønø) and an InnoExplorer grant (Grant 1046‐00023 to Gertrud Malene Hjortø).

## Supporting Information

Additional supporting information can be found online in the Supporting Information section.

## Supporting information


**Supporting Information** Figure S1. FACS analysis of the number of migrated cDCs formulated with C21TP to the popliteal lymph node following footpad injection.

## Data Availability

The data that support the findings of this study are available from the corresponding author upon reasonable request.
