# Supplementary material for: An Exploratory Study of the Impact of a CCL21‐Derived C‐Terminal Peptide on Dendritic Cell Lymph Node Homing
Source: J Immunol Res. 2026 Jan 5;2026:8833079. doi: 10.1155/jimr/8833079 (PMC12771625; doi:10.1155/jimr/8833079)
Supplement: Supplementary file 1 — Supporting Information Figure S1. FACS analysis of the number of migrated cDCs formulated with C21TP to the popliteal lymph node following footpad injection. [file JIMR-2026-8833079-s001.docx]

**Supplementary Material**

**Supplementary Figure 1. C21TP enhances the homing of dendritic cells to draining lymph nodes.** CD45.1^+^ αDC1s formulated with C21TP or PBS were injected into the footpad of CD45.2^+^ recipient mice. After 24 hours, the popliteal draining LN were isolated to quantify the number of migrated CD45.1^+^ cDCs (left) and endogenous APCs (CD45.2^+^ MHC-II) (right). *Statistics*: Data are presented as mean ± SD (n=2-8 individual pLN per treatment group. One experiment. Ordinary one-way ANOVA and Tukey´s multiple comparison test. p: p-values *: p < 0.05.
